# Endothelin Receptor Antagonists for the Treatment of Hypertension: Recent Data from Clinical Trials and Implementation Approach

**DOI:** 10.1007/s11886-025-02262-3

**Published:** 2025-07-02

**Authors:** Revathy Manickavasagar, Anoushka Krishnan, Omar Azzam, Markus P. Schlaich

**Affiliations:** 1 Department of Nephrology & Transplantation, Royal Perth Hospital, Perth, Australia; 2 Dobney Hypertension Centre, Medical School – Royal, Perth Hospital Unit and RPH Research Foundation, The University of Western Australia, Level 3, MRF Building, Rear 50 Murry St, Perth, WA 6000 Australia; 3 Department of Cardiology, Royal Perth Hospital, Perth, Australia

**Keywords:** Endothelin-1, Endothelin receptor antagonist, Hypertension, Pharmacotherapy, Blood pressure, Aprocitentan

## Abstract

**Purpose of Review:**

The endothelin system is a highly relevant component of the pathophysiology of hypertension, which is currently unopposed by existing treatment approaches. We examined the role of dual endothelin receptor antagonists in the treatment of resistant hypertension.

**Recent Findings:**

The recent PRECISION trial demonstrated significant blood pressure lowering effect with the use of the dual endothelin receptor antagonist aprocitentan in the treatment of resistant hypertension. Aprocitentan was shown to be particularly effective in patients over 75 years of age, African-American patients, and patients with diabetes and advanced CKD. There was also a decrease in proteinuria. Aprocitentan was well tolerated and the risk of fluid retention can be mitigated by close clinical monitoring and titration of diuretic therapy.

**Summary:**

Aprocitentan presents a novel treatment option for resistant hypertension, with particular efficacy noted in patient cohorts who have historically been challenging to achieve blood pressure targets in.

## Introduction

Hypertension is the most common chronic condition and leading risk factor for cardiovascular disease worldwide. It is a major contributor to morbidity and mortality globally. Resistant hypertension (RH) is estimated to affect 10–15% of those treated for the condition [1]. It is well recognised that resistant hypertension increases the risk of adverse cardiovascular outcomes, chronic kidney disease (CKD), stroke and reduced health-related quality of life [2–5]. In addition, features of hypertension-mediated organ damage (HMOD) such as carotid intima-media thickening, left ventricular hypertrophy, albuminuria and hypertensive retinopathy, are more common and more pronounced in patients with RH [6].

RH is defined as blood pressure (BP) above the recommended target range, despite treatment with maximally tolerated doses of at least three anti-hypertensive drug classes, including renin-angiotensin system blockade (angiotensin-converting enzyme inhibitors or an angiotensin II receptor blocker), a long-acting dihydropyridine calcium channel blocker and a diuretic. While somewhat controversial, patients with controlled blood pressure who require at least 4 antihypertensive medications are also commonly included in this category [7].

To confirm true RH, it is crucial to exclude factors such as inaccurate BP, white-coat effect, medication non-adherence and clinical inertia [7–9]. Utilisation of out-of-office measurements, such as ambulatory blood pressure monitoring (ABPM), is valuable in excluding white-coat hypertension and other inaccuracies related with office readings [8] are now considered mandatory for the diagnosis of RH. Those who have RH should generally be assessed for secondary causes of hypertension such as chronic kidney disease, primary hyperaldosteronism, obstructive sleep apnoea, renovascular hypertension, endocrinopathies, interfering medications, and others [7].

Common characteristics of RH include older age, male gender, African American race [10], obesity, higher baseline BP readings (particularly systolic), obstructive sleep apnoea, diabetes mellitus and the presence of established hypertension mediated organ damage such as left ventricular hypertrophy and CKD [7, 11, 12].

The pathophysiology of hypertension is complex, multifactorial and remains incompletely understood. Key components have been demonstrated to include excess sodium absorption and volume expansion, activation of the renin–angiotensin–aldosterone system and increased activity of the sympathetic nervous system [13]. Accordingly, the mainstay of pharmacological treatment targets various aspects of the renin–angiotensin–aldosterone system (renin inhibitors, angiotensin converting enzyme inhibitors, angiotensin receptor blockers, aldosterone antagonists), calcium channels (dihydropyridine and non-dihydropyridine calcium channel blockers), fluid retention and sodium homeostasis (various classes of diuretics), adrenergic receptors (alpha and beta blockers), centrally acting sympatholytic agents and vasodilatory pathways [7, 13]. However, despite the availability of powerful pharmacological options targeting established pathways in the pathophysiology of hypertension, a significant portion of patients with hypertension do not achieve target readings [14]. Failure to control BP with currently available drugs, assuming adherence with prescribed medication, suggests that other relevant pathophysiological pathways remain unopposed by current approaches. Availability of newer antihypertensive drug therapies with innovative mechanisms of action would be a valuable addition to current treatment strategies [15].

## The Endothelin System

Since the discovery of endothelin (ET)−1 by Yanagisawa et al. in 1998, the endothelin system has been recognised as a complex network, with its dysregulation contributing to a wide variety of pathologies [16]. The endothelin system is emerging as an important mediator in hypertension, with the endothelin receptor antagonists representing a promising target for new antihypertensives.

ET-1, a 21 amino acid peptide, is the most abundant and potent [17] of the three (ET-1, ET-2 and ET-3) endothelin isoforms in the human cardiovascular system [18, 19]. The major source of ET-1 is the vascular endothelial cell, where it is generated continuously to maintain vascular tone and blood pressure, as well as in response to various stimuli [18, 19]. ET-1 is also generated in other organs such as the heart and kidneys, in addition to a variety of cells including vascular smooth muscle cells, cardiomyocytes, fibroblasts, macrophages, neurons, and epithelial cells in the lungs and kidneys [18, 19]. The production of ET-1 is regulated by various stimuli including hypoxia, tumour necrosis factor (TNF), insulin, norepinephrine, angiotensin II, nitric oxide, naturetic peptides and free radicals [6, 18, 20].

ET-1 is a potent vasoconstrictor with a long half-life, exhibiting both paracrine and autocrine actions, which may account for its low levels detected outside the vasculature [6, 21]. The effects of ET-1 are mediated via two G protein coupled receptors, ET_A_ and ET_B_ (ET_B1_ + ET_B2_) receptors, with equipotent affinity to ET-1 [22]. ET_A_ and ET_B_ receptors are located in the vascular smooth muscle cells and endothelial cells. The ET-1 mediated stimulation of these two receptors, can result in different and opposing effects, and may vary depending on the distribution of the receptors in particular organs [17].

The binding of ET-1 to the ET_A_ and ET_B_ receptor in the vascular smooth muscle promotes vasoconstriction, in addition to inflammation and mitogenic cell growth response [23, 24]. Elsewhere, ET_A_ receptor engagement also promotes salt and water retention, inflammation and fibrosis. While both ET_A_ and ET_B_ receptors are found on vascular smooth muscle cells and mediate vasoconstriction, the ET_B_ receptor is also distinctively situated on the endothelium. Here, the ET_B_ receptor engagement promotes vasodilation mediated by the production of vasodilator substances, such as nitric oxide and prostaglandin-I [14]. In addition, endothelial ET_B_ stimulation promotes clearance of ET-1 from the circulation, sodium excretion and inhibits vascular inflammation and fibrosis [25, 26]. ET_B_ receptors in the collecting ducts of the kidneys play a crucial role in promoting naturesis [14, 27, 28]. In this manner, ET_B_ receptors are crucial in acting as a counter-regulatory pathway to limit ET_A_ receptor mediated vasoconstriction [26, 29]. However in pathophysiological conditions such as hypertension and pulmonary hypertension, ET_B_ may be upregulated on vascular smooth muscle cells leading to vasoconstriction and proliferation [14].

ET-1 has been implicated in the pathogenesis of various cardiovascular diseases such as hypertension, atherosclerosis, heart failure, pulmonary hypertension, coronary artery disease and renal disease [6] (Table 1). In the setting of pathophysiological conditions characterised by endothelial dysfunction, vasoconstriction and other pathophysiological effects of ET-1, such as cell proliferation, inflammation and fibrosis, are potentiated [17, 26]. Patients with moderate to severe essential hypertension have been shown to exhibit enhanced endothelial expression of the ET-1 gene [30]. ET-1 levels have been demonstrated to be elevated in atherosclerotic heart disease, heart failure and pulmonary arterial hypertension [31, 32], and have been shown to correlated with prognosis in the latter two [31, 33, 34]. ET-1 has also been shown to be involved in myocardial hypertrophy and remodelling occurring during heart failure, with activation of inflammatory and fibrotic processes, and ensuing cytokine overexpression, such as TNF alpha, interleukin (IL)−1 and IL-6 [33, 35]. In addition, overexpression of ET-1 and receptors have been associated with other conditions such as chronic kidney disease and diabetes mellitus [36].

**Table 1:** Major actions of endothelin-1 and associated pathology

Target	Receptor	Effects	Associated Pathology
Cardiovascular System	ET_A_	Coronary artery vasoconstriction ANP production	Heart failure Fibrosis Inotropy Hypertrophy Arrhythmogenicity Spontaneous coronary artery dissection
Vascular System	ET_A_	Sustained vasoconstriction, growth & proliferation Matrix production, Inflammation	Hypertension Atherosclerosis
Pulmonary Artery	ET_A_ & ET_B_	Proliferation, pulmonary vasoconstriction	Pulmonary arterial hypertension
Adrenal glands	ET_A_ & ET_B_	Increased aldosterone production & secretion	Hypertension
Kidneys	ET_A_	Vasoconstriction	Hypertension
		Decreased GFR	Diabetic nephropathy
	ET_B_	Diuresis Natriuresis	Glomerulonephritis Polycystic Kidney Disease
Macrophages	ET_A_	Inflammation	Atherosclerosis
Fibroblasts	ET_A_	Fibrosis	Fibrosis
Central Nervous System	ET_A_ ET_B_	Vasoconstriction ?Increased AVP production	Ischaemic/haemorrhagic stroke

*ANP* anti-natriuretic peptide, *ET*_*A*_ endothelin receptor type A, *ET*_*B*_ endothelin receptor type B, *GFR* glomerular filtration rate, *AVP* arginine vasopressin

Blockade of the endothelin system may offer additional benefits by alleviating other pathophysiological pathways involved in hypertension. For example, pre-clinical and clinical evidence demonstrates that ET-1 is a mediator of aldosterone release and ET receptor blockade is associated with a reduction in plasma aldosterone levels [37, 38]. Furthermore, ET-1 has been demonstrated to have a stimulating effect on sympathetic nerve activity through activation of the ET_A_ receptor, which may contribute to the pathogenesis of hypertension via contributory overactivity of the sympathetic nervous system [24].

Until recently, the clinical use of ET antagonists has been limited to the treatment of pulmonary hypertension, where it results in clear benefits attributed to the vasodilatory and anti-hyperplastic effects in the pulmonary vascular bed [39]. However, despite increasing evidence that ET-1 is implicated in the pathophysiology of hypertension, there have been limited recent clinical trials demonstrating the efficacy of ET receptor antagonists as a treatment for systemic or resistant hypertension. The recent publication of the PRECISION trial, has however illuminated the potential clinical utilisation of this class of medications for the treatment of RH.

## Endothelin Receptor Antagonists (ERAs) in Hypertension

ET-1’s potent and long-lasting vasoconstrictor and pressor actions have been implicated in the pathogenesis of hypertension and heart failure. Early salt sensitive animal studies demonstrated the BP lowering effects of this class of ERAs [40–42], leading to increasing numbers of experimental and clinical data evaluating the use of ERAs in hypertension and RH [6]. The therapeutic effects depend on whether there is selective ET_A_ receptor engagement or dual ET_A_ and ET_B_ receptor blockade [43].

Krum et al.’s first human study of ERAs in 1998 [44], a randomised controlled trial comparing the effects of bosentan to placebo in patients with primary hypertension, provided promising results. Bosentan is a highly specific, orally active non-selective ET_A_ and ET_B_ receptor antagonist. 293 patients with mild to moderate essential hypertension were randomised to receive one of four oral doses of bosentan (100, 500 or 1000 mg once daily or 1000 mg twice daily), placebo, or the angiotensin-converting-enzyme inhibitor enalapril (20 mg once daily) for four weeks. When compared to placebo, bosentan demonstrated a significant reduction in diastolic blood pressure, with a daily dose of 500 or 2000 mg (an absolute reduction of 5.7 mmHg at each dose) which was comparable to the reduction with enalapril (5.8 mmHg). However, the associated liver dysfunction and fluid retention observed in the bosentan exposed individuals posed a significant barrier to clinical utilisation.

Nakov et al [45] then investigated the effects of darusentan, a moderately selective ET_A_ antagonist, in a cohort with moderate hypertension. This randomised, double-blind, placebo-controlled parallel group comparison found that the highest dose of 100 mg daily of darusentan led to a sustained reduction of approximately 10 mmHg in BP over the six week treatment period compared to placebo. Again, adverse effects were significantly higher in the treatment group, with peripheral oedema, headache and flushing noted. Given the side effect profile of ERAs, this led to subsequent clinical trials focusing on the possible efficacy of darusentan in treating RH, where BP remains uncontrolled despite the use of standard triple antihypertensive treatment.

The DORADO trial [46] was a multi-center international trial which recruited 379 patients with RH. This study compared different doses of darusentan (50 mg, 100 mg, 300 mg daily) to placebo over 14 weeks. Darusentan achieved its co-primary end point of reducing mean systolic and diastolic clinic BP at week 14 of treatment compared to placebo (9/5 mmHg for placebo, 17/10 mmHg for 50 mg, 18/10 mmHg for 100 mg and 18/11 mmHg for 300 mg, p value < 0.0001). ABPM confirmed superior BP lowering efficacy of darusentan and showed a less pronounced placebo effect. This study suggested darusentan effectively lowered BP by an additional 10 mmHg in patients with RH who were already taking multiple antihypertensives at recommended doses. Of note, patients with diabetes and CKD, two frequent comorbidities of RH, experienced the most significant benefits from darusentan in achieving the recommended BP targets. Darusentan was associated with fluid retention and hypothetical risk of exacerbation of heart failure, although oedema was responsive to diuretic treatment. There were no significant adverse effects on kidney function in the combined darusentan treatment groups, and a significant reduction in urinary albumin excretion by 60% was observed.

This study was followed by the DORADO-AC trial [47], which studied 849 patients with RH who received either darusentan (50, 100 or 300 mg daily), placebo, or the active control, the centrally acting alpha-2 receptor agonist guanfacine 1 mg daily. Duration of treatment and co-primary end points were the same as the preceding DORADO trial. Again, there was a reduction in mean clinic systolic and diastolic BP at week 14, with a reduction of 15/10 mmHg with darusentan, which was significantly greater than that observed with guanfacine (12/6 mmHg). Notably, the placebo group also had a substantial decrease of 14/8 mmHg in mean clinic BP, particularly after 8 weeks of treatment, which could not be explained. While subsequent post hoc, time weighted analysis with data from ABPM, demonstrated superior reductions in mean systolic and diastolic BP of the darusentan compared with placebo and guanfacine groups, this difference was not statistically significant. The placebo response in this study led to darusentan not achieving the pre-specified co-primary endpoint. Side effects in the darusentan group were fluid overload, mainly in the first 6 weeks of treatment commencement, leading to heart failure in one patient.

The most recent and pivotal study investigating the potential benefits of ERAs in RH is the Phase 3 PRECISION trial [48]. PRECISION investigated aprocitentan, an active metabolite of macitentan, and a dual, combined ET_A_ and ET_B_ receptor antagonist which potently inhibits binding of ET-1 to ET_A_ and ET_B_ with an inhibitory potency ratio of 1:16 (ie stronger inhibitory effect on ET_A_ than on ET_B_ receptors) [26]. Dual ET_A_ and ET_B_ receptor blockage may be advantageous as it has been associated with a lower risk of fluid retention and vascular leakage compared with selective ET_A_ blockade, which causes nonselective vasodilation and vasopressin release due to overstimulation of ETB receptors [49]. Aprocitentan has a long half-life of 44 h, favourable safety profile and potentially has synergistic effects with renin angiotensin aldosterone system inhibitors and calcium channel blockers in animal studies [50].

PRECISION was an international multi-center, blinded, randomised parallel-group, phase 3 study which adopted a novel three-part design to assess the short term and sustained long-term effects of aprocitentan on BP in those with RH. The study period consisted of three sequential parts: Part 1 was a 4-week double-blind period with aprocitentan 12.5 mg, 25 mg or placebo (1:1:1 ratio); Part 2 was a 32-week single-blind period with aprocitentan 25 mg; and Part 3 was a 12-week randomised withdrawal period with aprocitentan 25 mg or placebo (1:1 ratio).

A total of 1965 individuals were screened for RH, defined as unattended automated office systolic blood pressure (uAOBP) of ≥ 140 mmHg, despite ≥ 3 anti-hypertensives from different classes including a diuretic. This screening period enabled increased sensitivity of diagnosis and treatment efficacy, by comprehensively excluding secondary causes of hypertension and adopting a higher uAOBP threshold for RH definition. Of those screened, 730 individuals were randomised, with the most common cause for exclusion (44.4% of all screened patients) being failure to meet the RH inclusion criteria, demonstrating the high portion of pseudo-resistant hypertension amongst patients referred for RH [15].

All 730 eligible patients were commenced on standardised background therapy (SBT), a single-pill combination of maximally tolerated doses of a calcium channel blocker (amlodipine), an angiotensin receptor blocker (valsartan) and a diuretic (hydrochlorothiazide) at fixed doses of 5/160/25 mg or 10/160/25 mg. To minimise a placebo response, a 4 -week single-blind run-in phase was incorporated, when patients received a placebo in conjunction with SBT. At the time of screening, 63% of all patients who were randomised were prescribed four or more antihypertensive agents, and all patients previously on a beta-blocker continued it for the duration of the study.

**Fig. 1:**
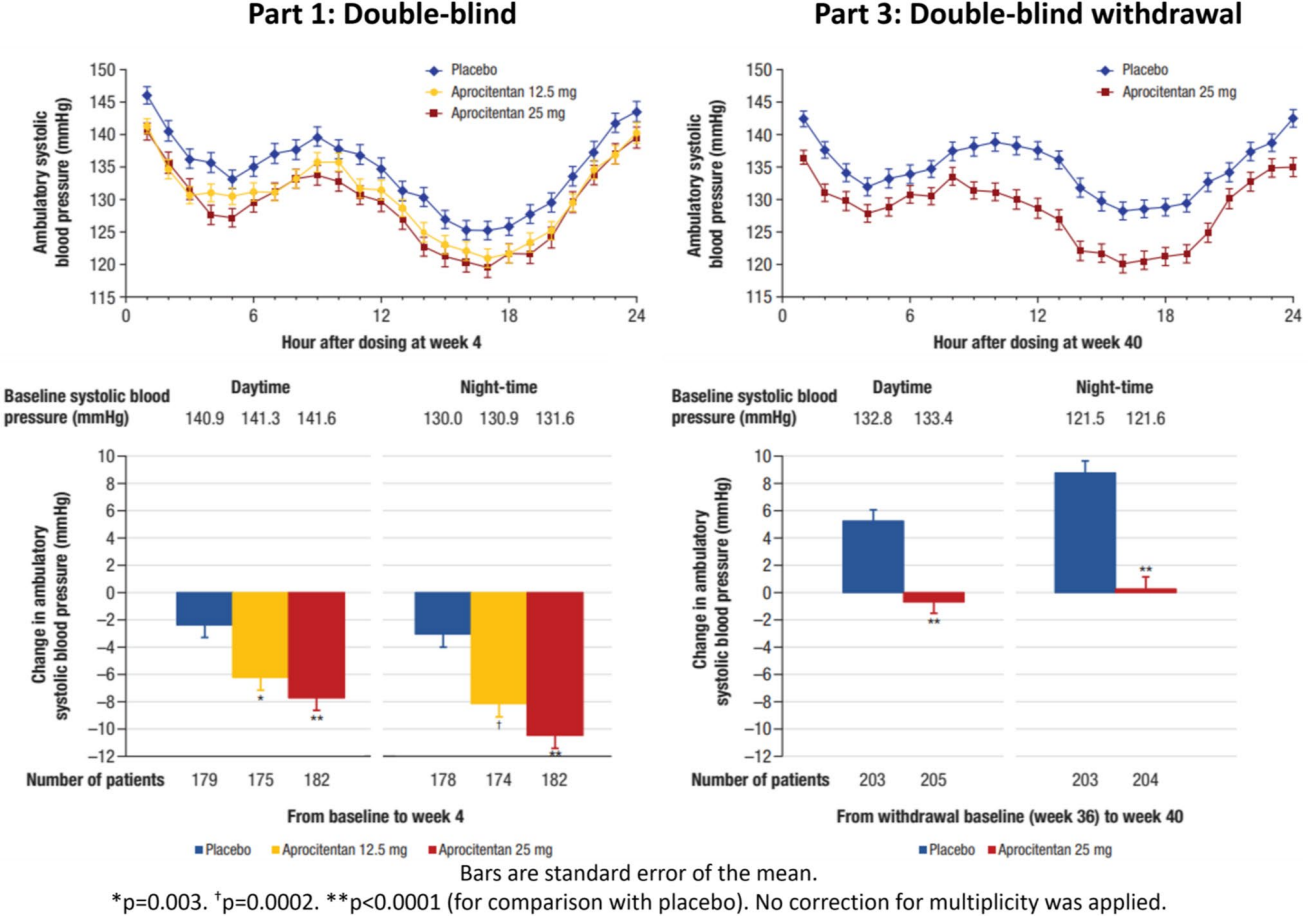
Effects on 24-h ambulatory BP. Changes in ambulatory BP in response to placebo (*blue*), aprocitentan 12.5 mg (*yellow*), and aprocitentan 25 mg (*red lines and bars*) after the 4-week double blind phase (Part 1) and after double-blind withdrawal (Part 3). 24-h profiles (upper panels) demonstrate BP reduction with both doses of aprocitentan compared to placebo across the 24-h period. Absolute BP changes depicted in the lower panels demonstrate a significant dose dependent effect, most pronounced during night-time (lower left panel). After re-randomization to placebo or continued aprocitentan 25 mg BP rose significantly in those receiving placebo, whereas BP was unchanged in those maintained on aprocitentan 25 mg (lower right panel). (Reprinted from: Schlaich MP, et al. Lancet 2022;400:1927–37, with permission from Elsevier) [48]

At 4 weeks, both aprocitentan doses (12.5 mg and 25 mg daily) were associated with statistically significant absolute reduction in unattended automated systolic and diastolic office blood pressure, -15.3/-3.9 mmHg and -15.2/-4.5 mmHg respectively, when compared with placebo. The difference in office SBP and DBP between the aprocitentan and placebo groups remained significant up to week 48, supporting long term-efficacy of aprocitentan. These findings were further supported by the ABPM data and substantial nocturnal BP reduction, a factor recognised as a stronger predictor of cardiovascular mortality in comparison to other BP markers [51] (Fig. 1).

**Fig. 2:**
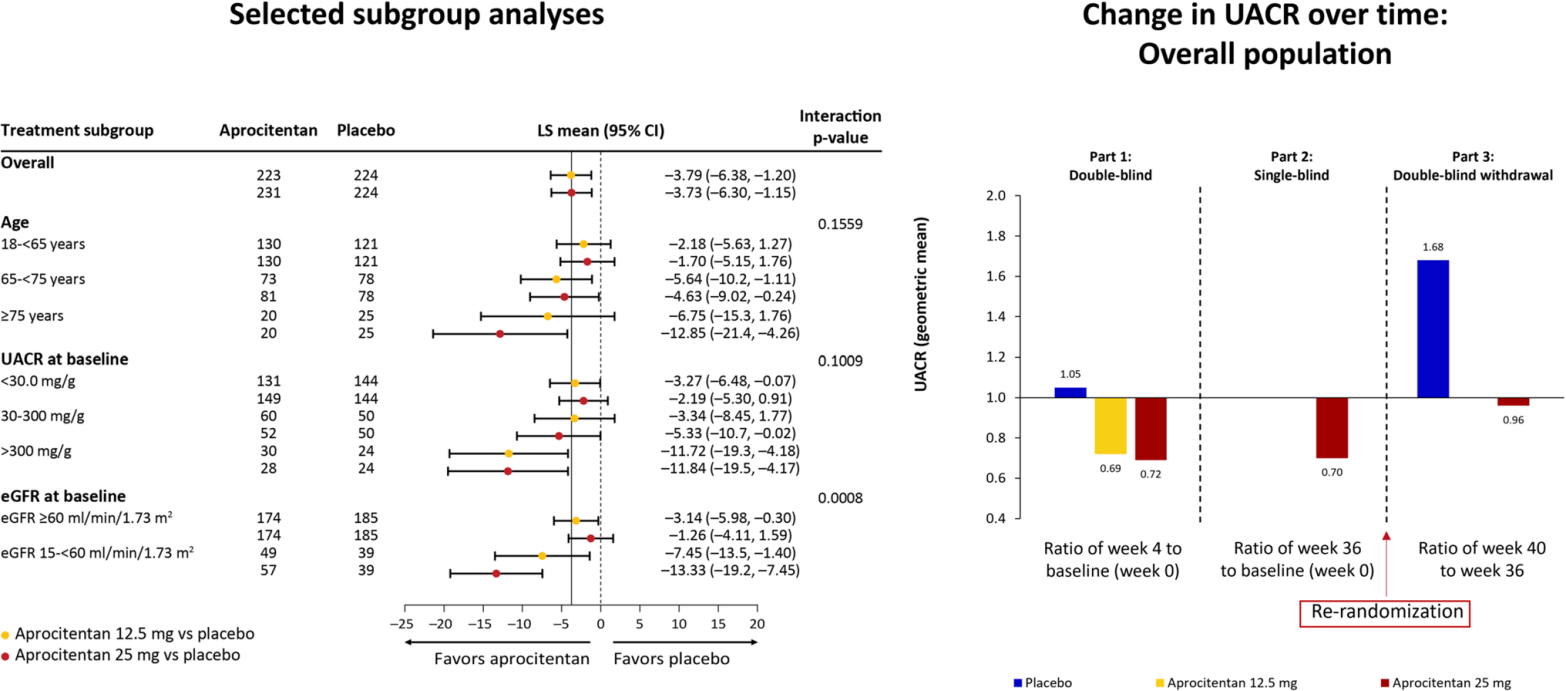
Subgroup analysis and effects on urinary albumin excretion (UACR). Subgroup analysis revealed that aprocitentan at each dose seems particularly effective in patients above the age of 75 years, those with UACR > 300 mg/g, and in patients with an eGFR of < 60 ml/min/1.73 m.^2^ (Forrest plot left panel). The impact on UACR is summarized in the right panel demonstrating significant and clinically meaningful reduction in UACR with both doses after the double-blind phase. In the single-blind phase the 30% reduction in UACR was maintained. After re-randomization UACR rose in those on placebo, but remined unchanged in those on continued aprocitentan 25 mg. (Reprinted from: Schlaich MP, et al. Lancet 2022;400:1927–37, with permission from Elsevier) [48]

The aprocitentan associated blood pressure reduction appeared particularly pronounced in trial participants with higher cardiovascular risk profile, older patients (≥ 75 years), those with macro-albuminuria (urine albumin-creatinine ratio > 300 mg/g), and those with stage 3–4 CKD 3 (estimated glomerular filtration rate of 15 to < 60 ml/min/1.73m^2^). These results are promising given characteristics such as advanced age, CKD, diabetes and African American ethnicity are generally associated with challenging BP control, and perhaps ERAs are the missing link in optimising their RH treatment (Fig. 2). There was no observable difference between patients with or without beta blocker treatment at screening.

Fluid retention occurred more frequently with aprocitentan compared to placebo, with the incidence demonstrating a dose-dependent pattern in Part 1, with 9.1%, 18.4% and 2.1% for patients on aprocitentan 12.5 mg, 25 mg and placebo respectively. The rate of fluid retention in Part 2 was indistinguishable to the aprocitentan 25 mg daily arm in Part 1. Oedema was mild to moderate and could be managed by additional diuretic therapy. Eleven participants required hospitalisation for heart failure, 10 of whom where on aprocitentan, at least half had a history of heart failure with or without CKD at baseline, and all had T2DM. Nevertheless, the overall less pronounced fluid retention observed with aprocitentan treatment may be partly accounted for by the more balanced ET_A_ and ET_B_ receptor antagonism, compared with the ET_A_ selective antagonists previously studied, in addition to all patients in the PRECISION trial receiving the diuretic hydrochlorothiazide as part of their SBT.

There are a number of notable strengths of the PRECISION trial. Unlike previous studies, PRECISION recruited patients representative of the population commonly affected by RH, with a significant number of participants also having diabetes, cardiovascular disease, cerebrovascular disease, chronic kidney disease and obesity. In addition, 37% of the participants from the USA were Black of African American, who are established to be at increased risk of resistant hypertension [10].

The utilisation of higher office BP threshold (SBP ≥ 140 mmHg) to define resistant hypertension in the study, as opposed to the guideline-reported value of SBP ≥ 135 mmHg, enhanced the sensitivity of the study to treatment effects. In addition, the 4-week run in period with treatment with SBT, helped minimise a potential placebo effect. Although the observed BP reduction in the placebo cohort likely indicates there is more to hypertension than pathophysiology and pharmacology [52]. The single-blind 32 week use of aprocitentan ensured any high risk patients were not denied a potentially beneficial treatment [52]. The key secondary endpoint of change in office SBP from withdrawal of aprocitentan at week 36–40 (Part 3) provided insights into the sustainability and reversibility of any change in BP. White coat hypertension, office blood pressure measurement errors and the potential influence of placebo was further reduced by the consistent utilisation of 24 h ABPM across the entire duration of the study.

Aprocitentan’s extended half-life of ≥ 44 h and low drug interactions could conceivably compensate for reduced medication adherence, however investigators ensured medication administration with pill counting, observation of pill administration prior to ABPM and examination of urine samples to detect the background medication intake. In addition, aprocitentan was overall well tolerated. While unsurprisingly there were increased rates of fluid overload in those exposed to aprocitentan, this could be reduced with early identification of at risk patients, such as those with a background of heart failure and CKD, close monitoring in the first 4 weeks of treatment, and early administration of diuretic therapy. Encouragingly, the increased signal in those with advanced age, proteinuria and CKD indicate subgroups who may have enhanced benefits.

## Clinical Perspective and Implementation Strategies

Resistant hypertension remains a challenge to treat with current available therapies, and is associated with significant end organ complications. Current validated guidelines in the treatment of RH are centred on combination therapy with three antihypertensives at maximum tolerated doses: angiotensin converting enzyme inhibitors or angiotensin receptor blockers targeting the overactive renin–angiotensin–aldosterone system, calcium channel blockers targeting transmembrane influx of calcium ions into vascular smooth muscle cells, and diuretics targeting salt and volume excess.

The MRA spironolactone is widely considered the preferred fourth line treatment for RH, largely based on the findings of the PATHWAY-2 [53] study, however their utilisation may be restricted due to associated hyperkalaemia, reduction in eGFR and off target anti-androgen effects including gynaecomastia and erectile dysfunction [53]. Alpha- and beta blockers are additional potential antihypertensives, however have been shown to be less effective in RH compared with spironolactone [53].

The findings from the PRECISION trial presents aprocitentan as a novel treatment option of RH with attestable safety, efficacy and durable effect over a 12 month period. As such, aprocitentan was approved by the Food and Drug Administration (FDA) at a dose of 12.5 mg in the USA in 2024 for the treatment of hypertension, by the European Commission (EC) in 2024 for both 12.5 mg and 25 mg for patients with resistant hypertension tolerating the 12.5 mg dose and in need of tighter blood pressure (BP) control, and by the Medicines and Healthcare products Regulatory Agency (MHRA) in the United Kingdom in 2025 for the same indication as in Europe. Aprocitentan was the first new oral anti-hypertensive to be approved by the FDA in almost 40 years [43].

While the FDA initially had put aprocitentan under the requirement for the Risk Evaluation and Mitigation Strategy (REMS) program, the FDA subsequently determined that the benefits of aprocitentan outweigh the risks of embryo-fetal toxicity and that the updated labeling is sufficient for conveying the necessary safety information. Consequently, The US FDA has fully released aprocitentan from its REMS requirement and has minimized the burden on the healthcare delivery system of complying with the REMS.

Aprocitentan represents a complementary approach to the treatment of RH targeting the currently unopposed but highly relevant pathophysiological pathway of the endothelin system. Additionally, interactions between the endothelin system and other contributory pathways may be potentially synergistic and complementary when opposed, possibly representing a missing link in the treatment of RH. ET-1 has a sympatho-excitatory effect, contributing to basal sympathetic vasomotor tone and baroreflex dysfunction. Hence the sympatholytic and baroreceptor-buffering actions of aprocitentan likely contributes to its effect on night-time BP, which may have advantageous prognostic implications given the strong association between night-time BP and cardiovascular risk [54, 55]. Similarly, ET-1 has multifaceted effects on the RAAS including stimulation of aldosterone [56], with aprocitentan being associated with decreased plasma aldosterone levels in those with RH without impacting potassium levels [10].

PRECISION highlighted the importance of excluding pseudohypertension, with 44% of screened patients improving on triple therapy antihypertensives. Study participants shared characteristics typical of patients with RH, including diabetes, cardiovascular disease, CKD and obesity. Encouragingly, aprocitentan demonstrated particular benefit in those who have generally been more challenging to treat, with more pronounced BP lowering effect observed in the elderly, Black and African-Americans, and those with CKD and diabetes. ET-1 production has been shown to be increased in patients with other risk factors for RH such as obesity, diabetes and CKD, hence this may partly account for their notable BP reduction.

PRECISION demonstrated a significant reduction in urine albumin-creatinine ratio of 38% and 31% for the 12.5 mg and 25 mg doses respectively, with placebo doses resulting in a 5% increase in the double-blind part 1 of the study. This antiproteinuric effect was again more prominent in patients with stage 3 and 4 CKD. Proteinuria is an independent risk factor for chronic kidney disease progression, cardiovascular disease, end-stage renal failure and all-cause mortality, and ERAs are emerging as potential therapeutic agents in chronic kidney disease and proteinuria. Increased expression of ET-1 has been implicated in progressive loss of renal function in those with diabetic nephropathy [57]. In addition, ET-1 exerts multiple pathophysiological effects, including injury to the vasculature, podocytes, tubulointerstitium and mesangium via multiple mechanisms including inflammatory cell infiltration, proliferation and fibrosis [57]. Furthermore, BP control is a crucial strategy to reduce proteinuria and delay progressive decline in renal function.

The phase 3 SONAR [58] trial demonstrated the value of atrasentan, a selective ET_A_ receptor antagonist, in protecting kidney function in carefully selected patients with type 2 diabetes and CKD who show an initial reduction in albuminuria with short-term endothelin receptor blockade in combination with renin–angiotensin–aldosterone system blockade. Long-term treatment with this ERA significantly reduced the risk of the primary composite renal outcomes of doubling of serum creatinine or end-stage kidney disease compared with placebo. Heerspink et al. also had promising results when examining the novel dual endothelin and angiotensin receptor antagonist in IgA nephropathy, with a statistically significant reduction in baseline urine protein-creatinine ratio in sparsentan compared with the aldosterone receptor antagonist irbesartan in adults with IgA nephropathy, with similar safety profile [59].

Black and African-American participants in PRECISION showed clinically significant and durable BP reduction with the addition of aprocitentan. It is well recognised that the prevalence of RH is higher in African Americans, and hypertension is more difficult to treat in this population [60, 61]. Hypertensive African-Americans have higher plasma and vascular ET-1 concentration than Whites [62, 63]and an increased prevalence of salt-sensitive and low-renin hypertension [64, 65], suggesting a critical role of ET. Additionally, primary hyperaldosteronism, which is associated with very high ET-1 plasma concentrations, is frequently observed in African-Americans [66]. Hence, ERAs may represent a new therapeutic approach with an additional mechanism of action particularly fitting the pathophysiology of hypertension in Black or African-Americans with RH and may have implications in the reduction of disparities in hypertension related outcomes [10].

Maintenance of fluid homeostasis is essential when commencing aprocitentan. The fluid retention observed in PRECISION was foreseeable, however occurred at lower rates when compared to previous ERA studies, which may be partly due to the dual ET receptor blockade and co-administration with the diuretic hydrochlorothiazide as part of the background SBT. Fluid retention primarily emerged in the initial 4 weeks of treatment in a dose dependent manner. Notably, the PRECISION participants who were prescribed loop diuretics prior to the study, namely those with CKD, were changed to hydrochlorothiazide as part of the SBT. Hence these participants were essentially made more vulnerable to fluid retention.

In this context, Kohan et al. called into question using 25 mg per day aprocitentan in individuals at high risk for fluid retention, such as patients with diabetes with resistant hypertension and chronic kidney disease or a history of heart failure and argued that doses lower than 25 mg per day of aprocitentan would achieve comparable blood pressure lowering with less fluid retention [67]. However, the ambulatory BP data from the PRECISION trial clearly indicated a dose response relationship in regard to BP lowering [48]. Furthermore, discontinuation of therapy due to oedema or fluid retention was rare and reported in seven patients on 25 mg of aprocitentan across the entire study period of 48 weeks, even though all 704 patients were on 25 mg of aprocitentan for 32 weeks during the single-blind part 2 of the trial [48, 68].

Nevertheless, prior to commencing treatment, one should ensure optimal fluid status in patients, particularly those with CKD, diabetes and heart failure. It may be preferable to start with the lower dose of 12.5 mg of aprocitentan with close monitoring of fluid status at initiation, and have a low threshold for intensifying diuretic treatment, especially in those with additional risk factors. The opportunity to combine ERAs with the now widely prescribed sodium-glucose transport protein 2 (SGLT2) inhibitors may further reduce proteinuria, provide greater renal protection and mitigate the risk of potential fluid retention. Reassuringly unlike previous ERAs, there were no episodes of hepatotoxicity associated with aprocitentan, and it can be combined with other classes of anti-hypertensives.

Trials assessing target organ damage, cardiovascular and renal outcomes are key to understanding the long term effects of ERAs. Additionally, direct comparisons between MRAs and ERAs may help personalise treatment and identify patient subsets who are more likely respond to ERAs [6]. With the expanding armamentarium of therapies improving cardiovascular outcomes and progressive kidney disease, including SGLT2 inhibitors, glucagon-like-peptide-1 (GLP1) receptor agonists and non-steroidal MRAs, it is exciting to see how ERAs can be incorporated.

## Key References

- Azzam O, Nejad SH, Carnagarin R, Nolde JM, Galindo-Kiuchi M, Schlaich MP. Taming resistant hypertension: The promise of novel pharmacologic approaches and renal denervation. Br J Pharmacol 2024;181:319–39.
  - This review article provides a comprehensive diagnostic and management approach of resistent hypertension.
- Dhaun N, Webb DJ. Endothelins in cardiovascular biology and therapeutics. Nat Rev Cardiol 2019;16:491–502.
  - In-depth review of endothelins in cardiovascular biology and possible therapeutic benefits targeting this system.
- Weber MA, Black H, Bakris G, et al. A selective endothelin-receptor antagonist to reduce blood pressure in patients with treatment-resistant hypertension: a randomised, double-blind, placebo-controlled trial. Lancet 2009;374:1423–31.
  - The pivotal DORADO trial evaluating the effects of variable doses of darusentan, a selective endothelin-receptor antagonist in resistent hypertension.
- Schlaich MP, Bellet M, Weber MA, et al. Dual endothelin antagonist aprocitentan for resistant hypertension (PRECISION): a multicentre, blinded, randomised, parallel-group, phase 3 trial. Lancet 2022;400:1927–37.
  - The first phase 3 clinical trial assessing safety and efficacy of Aprocitentan, a novel dual endothelin receptor antagonist in the treatment of resistant hypertension.
- Bakris GL, Lindholm LH, Black HR, et al. Divergent results using clinic and ambulatory blood pressures: report of a darusentan-resistant hypertension trial. Hypertension 2010;56:824–30.
  - The DORADO-AC trial comparing the effect of darusentan, a selective endothelin receptor antagonist to placebo and active control of guanfacine 1 mg daily, in resistant hypertension.

## Data Availability

No datasets were generated or analysed during the current study.
